# The use of a natural substrate for immobilization of microalgae cultivated in wastewater

**DOI:** 10.1038/s41598-020-64656-3

**Published:** 2020-05-13

**Authors:** Tomasz Garbowski, Mirosława Pietryka, Krzysztof Pulikowski, Dorota Richter

**Affiliations:** 1 Institute of Technology and Life Sciences, Falenty, Al. Hrabska 3, 05-090 Raszyn, Poland; 2 Wrocław University of Environmental and Life Sciences, Department of Botany and Plant Ecology, Grunwaldzki Square 24a, 50-363 Wrocław, Poland; 3 Wrocław University of Environmental and Life Sciences, Institute of Environmental Engineering, Grunwaldzki Square 24, 50-363 Wrocław, Poland

**Keywords:** Environmental sciences, Urban ecology, Biofilms, Environmental microbiology, Industrial microbiology

## Abstract

The methods of separation of microalgae has a significant impact in the economic aspects of their cultivation. In this study, pine bark was used as a substrate for immobilization of microalgal biomass cultivated in raw municipal sewage. The experiment was conducted in cylindrical photobioreactors (PBRs) with circulation of wastewater. Biomass was cultivated for 42 days. After that time, abundant growth of the biofilm with microalgae on the surface of pine bark as well as improvement of the quality of treated sewage were observed. The efficiency of removal of nutrients from wastewater was 64–81% for total nitrogen and 97–99% for total phosphorus. Moreover, the concentration of suspended solids in sewage was reduced, which resulted in a decrease in turbidity by more than 90%. Colorimetric analysis and Volatile Matter (VM) content in the substrate showed a decrease in the Higher Heating Value (HHV) and concentration of VM due to the proliferation of biofilm.

## Introduction

Cultivation of microalgae is increasingly popular in many industrial sectors. The biomass of these microorganisms and its bioproducts are used in pharmaceutical, food, feed, chemical, cosmetic industries and aquacultures^1–3^. The sectors that implement technologies of microalgae production are also renewable energy and biorefineries^4,5^. Currently, the most advanced technology for the production of microalgae is the use of photobioreactors (PBRs). Their advantages in comparison with open ponds are better control of the conditions of cultivation, reduced risk of contamination by other microorganisms (fungi, molds, bacteria, protozoa and microalgae), and operation at high biomass density^1,2,4,5^. Along with the many benefits of production of microalgae biomass, there are also several restrictions. The main issue is the cost of cultivation of this type of biomass. It is estimated that the cost of production of 1 kg microalgae biomass ranges between 20 and 200$^5^. Compared to the production of terrestrial plants biomass (€0.20 kg^−1^ for soybeans and €0.35 kg^−1^ for wheat and corn^2^), these are significant amounts. Such large financial outlays in microalgae production in PBRs are generated mainly by the preparation of cultivation medium, irradiation, harvesting of biomass, CO_2_ supplementation and mixing^5–7^. Among these factors, the costs of harvesting and separation of biomass may constitute up to 20–30% of the total production costs^7^. Therefore, the selection of a suitable method of separation of biomass is crucial in the economic aspect of cultivation^1^. The difficulties in harvesting microalgae are mainly related to their small size and large dispersion in the cultivation medium^6^.

One of the methods of separating the microalgae suspension is its immobilization in the form of a biofilm on a solid substrate^8,9^. This system significantly reduces the costs of cultivation in comparison with conventional methods of separation (flocculation, membrane filtration, centrifugation etc.) and facilitates the harvesting of biomass^3,6,7^. Biofilm in PBRs constitutes a structure composed mainly of colonies of bacteria and microalgae. Biofilm is formed by the secretion of extracellular polymeric substances (EPSs) that merge microorganisms^3,10^. The main producer of these substances are bacteria, and the more EPSs are extracted, the structure of biofilm will be more durable^11,12^. Among the most popular materials used as a substrate for the microalgae cultivation are artificially manufactured substrates from polyester, cotton or nylon fibers, as well as concrete and polystyrene^7,13,14^. However, natural substrates, which can be cheaper and easier to gain and even constitute waste materials, are not commonly used.

To reduce the costs of microalgae cultivation (for energy or biorefinery purposes), it is also possible to replace the synthetic cultivation medium with wastewater which is a rich source of nitrogen and phosphorus^9,15,16^. Microalgae use inorganic nitrogen and phosphorus for growth^17–19^ and due to their high resistance to pollution and the ability to easily adapt to environmental conditions they are able to proliferate in many types of wastewater^16^. Many researchers suggest using municipal, domestic, agricultural (containing nutrients from fertilizers^20^) and industrial wastewater, effluents from landfills and biologically treated sewage for cultivation of microalgae^3,14,16,21^. The use of biofilm and sewage in algae cultivation contributes not only to a significant reduction of costs of biomass production, but also to the removal of nutrients, heavy metals, suspended solids, as well as toxic organic compounds^10,17,22^.

The manuscript presents the results of a study in which a natural substrate was used for the immobilization and separation of microalgae. The substrate was pine bark and microalgae cultivation was conducted in a cylindrical PBR supplied with raw municipal sewage. It was examined whether the biofilm can develop on a substrate so far not used for this purpose, as well as what is the impact of this structure on the quality of treated sewage. The aim of the study was to direct attention to the possibility of using easily available, fully natural materials as substrates for the cultivation of microalgae. The effect of proposed solution will be to reduce the costs of production of biomass with a simultaneous benefit for the environment resulting from the wastewater treatment effect and safe use of the substrate together with the produced biofilm.

## Materials and methods

### Microalgae cultivation

Microalgae were cultivated in laboratory scale in two cylindrical PBRs, each consisting of a cylinder (1 m high and 0.15 m diameter) made of acrylic glass (PMMA). The cylinders were filled with pine bark (height 0.40 m) previously cleaned, rinsed with water and dried at 105 °C for 24 hours. The bark was crushed into a few centimeters pieces and secured in a polyethylene mesh at a distance of 0.20 m from the bottom of the cylinder, which prevented the clogging of outlet of the PBRs. The total weight of the bark used as a substrate was approximately 0.70 kg in two cylinders. Pine bark in the PBRs formed a packed bed with thickness approximately 0.4 m. It was decided to use bark from pine trees because of the large range of this species in Europe and ease of acquisition. High porosity and roughness of the substrates are also important in the development of biofilm^3,7^ and lignocellulose materials have additionally hydrophilic properties^23^. In addition, preliminary studies using artificial (PET bottles and mats with polymer fibers)^9^ and natural materials (bark of pine, birch, oak, beech, ash, wood chips and charcoal) have shown the presence of a clear biofilm (visible to the naked eye) containing microalgae only on the surface of the pine bark. The cultivation medium in the PBRs was raw municipal sewage previously filtered through a 4 mm sieve to remove larger solid particles that could damage the pumps.

**Figure 1:**
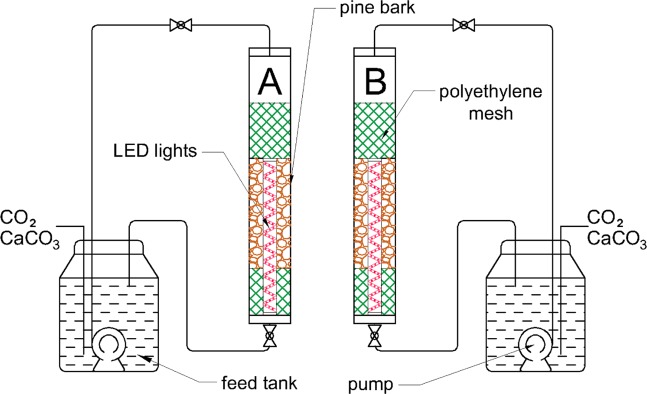
Scheme of cylindrical PBRs used for microalgae cultivation in the experiment.

The cylinders were closed on both sides, and the wastewater was supplied from two 25 dm^3^ tanks to the upper part of the PBRs. Thanks to the centrifugal pumps immersed in sewage, it was possible to transport the cultivation medium to a height of 1.50 m. The treated sewage was discharged from the bottom of the PBRs to the feed tanks. PBRs were operated in a closed sewage system with a flow rate of 1.50 dm^3^ min^−1^. The circulation of the sewage ensures a continuous nutrient supply, which results in better productivity than in batch reactors^3^. The scheme of the experimental set-up is shown in Fig. 1. In order to ensure optimal conditions for algae growth, a source of inorganic carbon in the form of CO_2_ and CaCO_3_ was introduced into wastewater. Thanks to this, a mixture easily absorbed by microalgae bicarbonates (HCO_3_^−^) is formed^6,24,25^. The proportions of these components were regulated so as to maintain a relatively constant pH value in the cultivation. The CaCO_3_ dose was calculated using the carbonate-calcium equilibrium nomogram for water^26^ based on the measurement of the alkalinity and current pH of sewage. The pH of wastewater was maintained at the level of 7.0–8.0, because at this pH, the largest amount of bicarbonate ions occurs in sewage. The culture was illuminated by LED light with intensity of 612 lux. LED lights were submerged in the sewage and placed in the central part of each cylinder. The artificial illumination of microalgae cultures provided control of Photosynthetic Photon Flux Density (PPFD), photoperiod and wavelength of light^27^. The applied LED tape with a length of 3 m contained diodes of red and blue light in a 5/1 ratio. The color of the supplied light was in the PAR range (400–700 nm)^1,27^ and the value of PPFD was 13.528 µmol m^−2^ s^−1^. The PBRs were illuminated with LED light during the night for 12 hours. During the day, microalgae used sunlight for photosynthesis. The internal light was applied in order to prevent light scattering on the walls of the PBRs, which may affect the growth of microalgae^28^. Moreover, each cylinder was twice inoculated by microalgae (V = 400 ml) from a separate cultivation conducted in suspension.

### Analysis of wastewater and substrate parameters

The cultivation of microalgae was conducted for 42 days at 25 °C. At that time, samples of treated wastewater were studied, with a 7-day frequency. The concentration of nitrogen (NO_3_^−^, NO_2_^−^, NH_4_^+^, N_organic_ and Total Nitrogen), phosphorus (PO_4_^3−^ and Total Phosphorus), turbidity and reaction (pH) were determined in the sewage. Nitrogen, phosphorus and pH were determined in accordance with accepted standards^29^. Phosphates were determined by spectrophotometric method with ammonium molybdate, whereas turbidity was measured by CyberScan TBD IR 1000 nephelometer. Samples for the determination of mineral forms of nitrogen and phosphorus were previously subjected to sedimentation. Total nitrogen and total phosphorus were determined together with the suspension, because the sewage was subject to mineralization process prior to these analyses. All measurements were also made for raw sewage.

In order to verify the growth of microalgae, microscopic observations of the bark surface with biofilm were conducted. Observations were carried out under Nikon Eclipse TE2000-S digital microscope equipped with a Nikon DS-Fi1 camera. Slide cover surface (18 × 18 mm) was considered the standard surface.

The so-called visible “calculation units” were counted (individual cells, coenobia, colony, 100 µm filament fragments were treated as “calculation units”). The calculations were conducted in order, along the parallel specimen lines, through moving the field of vision by one unit. The taxonomy of cyanobacteria and algae is based on^30^. Cyanobacteria and algae were identified according to the following studies^31–36^. The quantitative content of particular taxa was determined under the microscope using modified Starmach’s scale^37^, where 1 means individual occurrence of a given species (up to 10 calculation units on standard surface); 2 – from 11 to 50 units on standard viewing surface; 3 – from 1 to 5 calculation units in every field of vision; 4 –> 5 calculation units in every field of vision; 5 dominant or water bloom (occupying >50% surface of field vision).

The indirect parameters monitoring the growth of biofilm on pine bark were the content of Volatile Matter (VM) in the substrate as well as the changes in its Higher Heating Value (HHV). The HHV was measured using an Isoperibol Calorimeter Parr 6400. HHV and VM were tested for samples of crude pine bark and bark with proliferated biomass from both PBRs. The material for calorimetric measurements was pulverized, dried at 105 °C for 24 h and formed into pellets weighing approximately 1 g (3 pellets for each sample), which were subsequently combusted in a calorimetric bomb. Volatile Matter was measured by combustion of 2 g pulverized and dried material at 550 °C up to obtain a pure mineral fraction.

## Results and discussion

**Figure 2:**
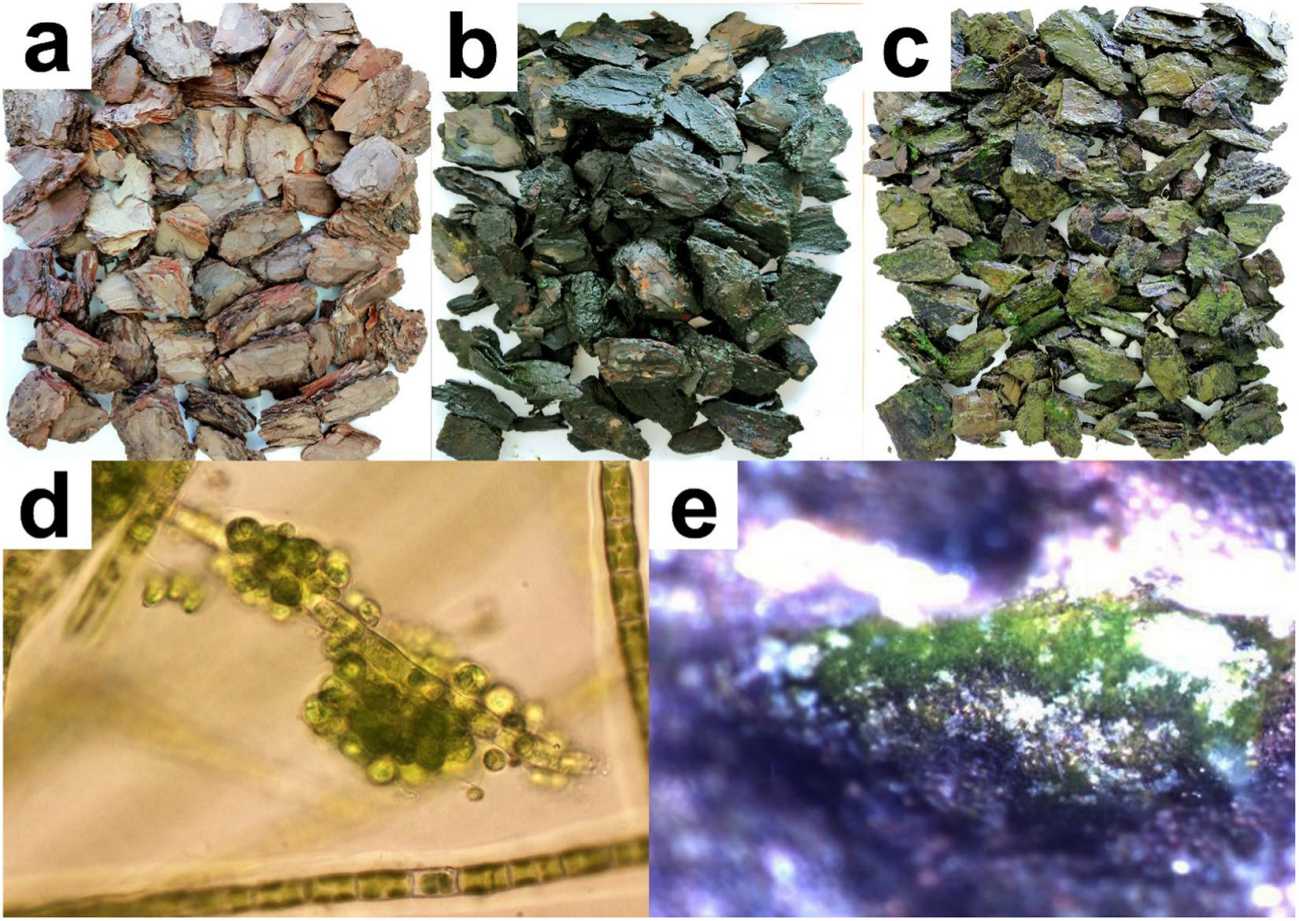
The presence of biofilm with microalgae on pine bark (**a**-*crude bark*, **b**-*bark from PBR-A*, **c**-*bark from PBR-B*, **d**-*filamentous and coccoid algae in biofilm*, **e**-*microscopic image of biofilm on the pine bark*) [phot. T. Garbowski, M. Pietryka, D. Richter].

The observations showed that the biofilm on the pine bark substrate started to develop after the first 7 days of the experiment. The important factor influencing the growth of biofilm was light^5^, introduced inside the cylinders of PBRs. Due to the limited penetration of external light through the pine bark into the central part of the PBRs, it was decided to introduce internal lighting of the cylinders. Thanks to this, the outer layers of the bark received daylight, whereas the inner layers of the bark were illuminated by artificial light during the night. The effect of this solution was the development of biofilm in the entire volume of cylinders. According to^38^, the effect of light hindering in PBRs with internal lighting can be reduced by using a stronger light source and reducing the distance between the light sources (e.g. greater packing of the LED tape in the reactor). In addition, the advantage of internal illumination is the possibility of expanding the dimensions of the reactor without loss of photosynthesis efficiency^38^ which is important when using PBRs on a large-scale. Slightly more abundant growth of microalgae on the pine bark was noted in reactor B (Fig. 2). These small differences in the growth of biomass could result from the differences in the efficiency of the use of sunlight by photoautotrophs during the day. According to^39^, most of the light energy is absorbed in the 2 mm layer of biofilm, hence the use of internal artificial light which shortens the light path and seems to be an effective solution to enhance photosynthesis. The biofilm formed in PBRs consists of consortia of microorganisms (diatoms, green algae, cyanobacteria, fungi, protozoa etc.) as well as detritus and mineral fraction^10,13,14^. Due to the symbiosis of bacteria and microalgae in the biofilm, it is possible to reduce the concentration of oxygen produced during photosynthesis, and thus protect microalgae against oxidative stress which is important especially in closed PBRs^3^. The presence of consortium of microalgae and bacteria in the biofilm increases the tolerance of the culture to changing environmental conditions and periodic nutrient deficiencies. Moreover, it improves the efficiency of nitrogen and phosphorus uptake from the cultivation medium^10^. The application of biofilm to microalgae cultivation causes their concentration, which reduces the costs of production of biomass, however, it makes it difficult to measure the amount of biomass^3,40^. In addition, biofilm limits the availability of light due to the phenomenon of self-shading and the presence of bacteria, which affects the synthesis of bioproducts^3,7^.

Table 1 shows the composition of species present in the biomass of cyanobacteria and microalgae populating pine bark in both PBRs. 11 taxa of cyanobacteria and algae were identified in the studied samples. Coccoid forms dominated in biofilm: *Chlorella* sp., *Oocistis* sp. and *Scenedesmus obliquus*. Filamentous forms were also abundantly present on bark surface, e.g. *Microspora quadrata*, *Ulothrix tenerrima* and *Tribonema minus*. Moreover, in PBR-B the study recorded abundant presence of small round epiphytic green algae growing on filamentous algae. Among the microalgae introduced as an inoculum, *Ulothrix tenerrima* Küzting (3), *Nitzschia palea* (Kützing) W. Smith (4), *Chlorella* sp. (4), *Oocistis* sp. (4), and other coccoid green algae (3) occurred. Other species presented in the biofilm came from the wastewater feeding reactors. Filamentous algae are a significant element of the biofilm. Their development strengthens the biofilm structure^14,41^ and contributes to stopping the pollution from sewage due to its cross-linked absorption surface^14^. Additionally, filamentous forms facilitate other species attachment (e.g. coccoid species) (Fig. 2d)^23^, which supports nutrient removal due to more effective biomass immobilization on the surface. Among the identified micro-algae occurring on pine bark, cyanobacteria and diatoms represented by *Nitzschia palea* were less numerous.

**Table 1:** Cyanobacteria and microalgal species present in the biofilm on a substrate of pine bark in PBRs A and B at the end of experiment.

Groups	Species	Bark PBR-A	Bark PBR-B
Cyanobacteria	*Leptolyngbya* sp.	—	2
	*Pseudanabaena limnetica* (Lemm.) Komárek	1	—
	coccoid cyanobakteria	1	—
Chlorophyta - filamentous species	*Microspora quadrata* Hazen	3	3
	*Ulothrix tenerrima* Küzting	3	2
Chlorophyta - coccoid species	*Chlorella* sp.	4	4
	*Oocistis* sp.	4	4
	epiphyte green algae	—	4
	*Scenedesmus obliquus* Tirph. Kützing	2	4
Bacillariophyceae	*Nitzschia palea* (Kützing) W. Smith	2	2
Xantophyceae	*Tribonema minus* Hazen	2	3

The combination of algae and bacteria properties allows the use of microbiological biofilm in bioremediation technologies^10^. Due to the high concentration of biomass in the PBR, it was possible to achieve high efficiency of removing nitrogen and phosphorus from sewage.

**Figure 3:**
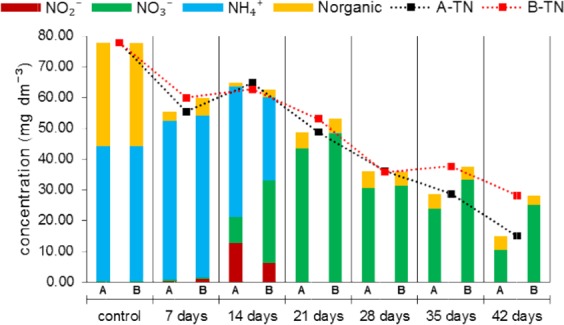
Changes in the concentration of nitrogen in sewage fed to PBRs A and B during the cultivation of microalgae.

Figure 3 shows the changes in the concentration of different forms of nitrogen in sewage treated in PBRs A and B. The growth of the biofilm with microalgae biomass caused a regular decrease in the concentration of Total Nitrogen (TN) in sewage from both PBRs. The reduction rate of total nitrogen in comparison to raw sewage in reactor A was 81%. According to^7,10,16^, the efficiency of TN removal from wastewater by biofilm in algal cultivation varies in the range of 80–97%. In reactor B a lower removal efficiency of TN (64%) was obtained. This may have resulted from differences in the efficiency of the photosynthesis due to the efficiency of the use of solar energy by photoautotrophs. Light affects the activity of intracellular enzymes responsible for the uptake and use of nutrients^27^. In the raw sewage (control), the dominant form of nitrogen was organic (N_organic_) and ammonium (NH_4_^+^) nitrogen. Part of N_organic_ was converted by the bacteria into ammonium ions (ammonification). As a result of this process, a decrease in the concentration of organic nitrogen and an increase in the concentration of ammonium nitrogen was observed on the 7^th^ day of the experiment. The different forms of nitrogen are introduced into the sewage as a result of leaching of pine bark. About 80% of the nitrogen leached form pine bark occurs in organic form^29^. This may affect the presence of organic nitrogen in the wastewater during the whole experiment. The coexistence of bacteria and microalgae in biofilm causes competition for ammonium nitrogen. Bacteria use NH_4_^+^ in the process of nitrification, while microalgae uptake these ions directly from sewage and build them into biomass^7,24^. On the 14^th^ day of the experiment, the process of nitrification was observed, in which ammonium ions are transformed into nitrites and nitrates under aerobic conditions. On that day, an increase in the concentration of TN was also observed, which was caused by re-inoculation of sewage in both PBRs by microalgae cultivated in the solution of NH_4_NO_3_. As a result of the introduction of microalgae suspension along with the cultivation solution, the concentration of NH_4_^+^ and NO_3_^−^ in wastewater increased. Ammonium nitrogen, accumulated by microalgae and oxidized by bacteria, became depleted, ipso facto over the following days of the experiment (21–42 days) the dominant form of nitrogen in the sewage was nitrates. This form of nitrogen is also used by microalgae for the synthesis of nitrogen compounds, however, it must be previously reduced in their cells to NH_4_^+^^3,24^. As a result of nitrates uptake by growing microalgal biomass, the concentration of TN in sewage continuously decreased. Taking into account the high efficiency of nitrogen removal from sewage by microalgae immobilized on solid substrate, this technology can be used, among other things, for the treatment of wastewater from Service Areas. This kind of sewage contains high concentrations of TN (up to 400 mg dm^−3^), mainly in the form of NH_4_^+^^42^.

**Figure 4:**
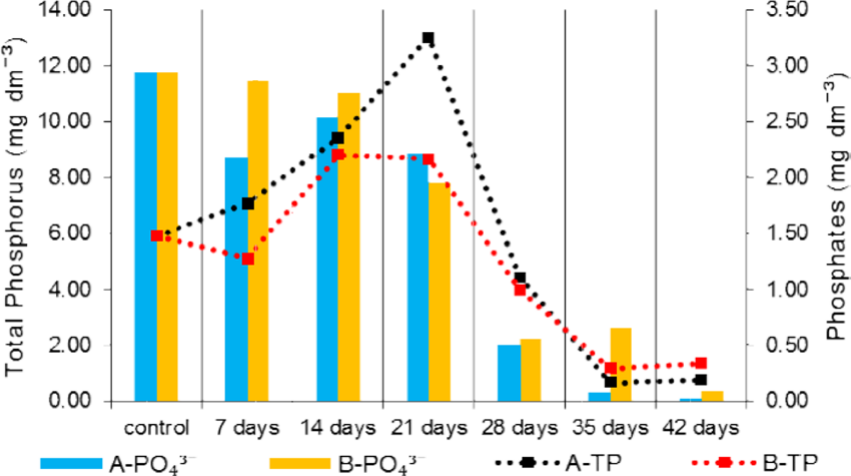
Changes in the concentration of Total Phosphorus (TP) and phosphates (PO_4_^3-^) in sewage fed to PBRs A and B during the cultivation of microalgae.

The available form of phosphorus for microalgae are phosphates (PO_4_^3−^)^6,17^ which are found in raw sewage and require removal. The microbial biofilm can reduce the concentration of Total Phosphorus (TP) by over 70% (even up to 97%)^7,10,16^. The concentration of TP is also influenced by the content of organic phosphorus, of which large amounts are also present in raw sewage. Figure 4 shows changes in the concentration of PO_4_^3−^ and TP in wastewater that feeds PBRs A and B. Phosphorus is one of the key components in the growth of microalgae, because 1 g of P can contribute to the development of approximately 1.70 kg of microalgae biomass^43^.

**Figure 5:**
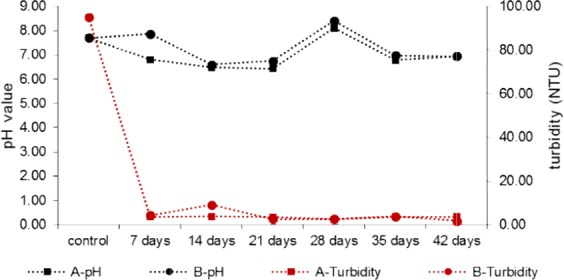
The value of pH and turbidity in sewage fed to PBRs A and B during the cultivation of microalgae.

In both PBRs, compared to raw sewage (control), a decrease in the concentration of phosphates was observed. The reduction of the concentration of PO_4_^3−^ was 99% for reactor A and 97% for reactor B. These results demonstrate that the uptake of this component by microorganisms in biofilm is intensive. In order to prevent precipitation of phosphates into sparingly soluble salts, pH adjustment is required. Phosphates at high pH can form salts with Al^3+^, Mg^2+^, Ca^2+^, Fe^3+^ ions and become unavailable to microalgae^22,24^. Due to maintaining the carbonate-calcium equilibrium in the PBRs by adding CO_2_ and CaCO_3_, the phenomenon of phosphate precipitation was limited. In contrast to phosphates, the concentration of TP increased until 21^st^ day of the experiment. The reason for the increase in the concentration of TP was the leaching of pine bark by flowing sewage. Phosphorus is one of the main components leached from pine bark. It is extracted both in the form of phosphates and organic phosphorus^44,45^. The study showed that after 1 day of leaching of pine bark with distilled water approximately 0.065 mg g_DW_^−1^ of TP was leached^29^. Phosphates were absorbed by microalgae, thus there was no increase in their concentration due to leaching of the bark. However, organic phosphorus is difficult to remove, hence its accumulation in the sewage flowing through the pine bark was observed. Between 21^st^ and 28^th^ day of the experiment, a significant reduction of the concentration of PO_4_^3−^ as well as TP was noted. During that time, an intensive growth of microalgae in the PBRs was observed, which favored the intensive uptake of PO_4_^3−^ ions. The concentration of organic phosphorus could be reduced due to precipitation and retention in the biofilm structure as well as adsorption on the surface of microalgae cells^6,14^. Furthermore, the EPSs secreted by microorganisms could serve as bioflocculants^1^, which in combination with filtration on a bed of pine bark could contribute to a significant reduction in the concentration of organic phosphorus. An increase in the pH value noted on the 28^th^ day of the experiment (Fig. 5), and the presence of Ca^2+^ ions could additionally intensify the process of phosphorus precipitation.

According to^3^, PBRs with biofilm give better results in removing pollutants from sewage than in the case with cultivation of microalgae in suspension, and biomass bound on the substrate can constitute over 72% of the total biomass produced. Moreover, hybrid PBRs (with microalgae-bacterial biofilm) demonstrate greater efficiency in the reduction of nutrients than PBRs operating in the monoculture system^10^. The optimal pH for the cultivation of microalgae ranges between 7 and 9^21^. This value of pH was also maintained in the conducted cultivation (Fig. 5). Inadequate pH of the cultivation medium can disrupt cellular processes and, as a consequence, cause the death of microalgae culture^46^.

The applied substrate from pine bark together with growing biofilm was characterized by high efficiency in removing suspended solids from the sewage. Already in the first week of the experiment, turbidity in the sewage was reduced by over 90% in both PBRs (Fig. 5). Pine bark like other biosorbents is able to retain various types of organic and inorganic pollution^47–49^. The bark also acts as a natural biofilter for solid particles^48^, thus contributes to the retention of microalgae biomass and wastewater treatment. The suspended particles were mechanically retained on the pine bark surface which confirms a significant decrease in turbidity of sewage after 7 days of the experiment. Pine bark in the cylinders of PBRs was firmly packed and the sewage flowed gravitationally through the bark that caused effective removal of suspension. Moreover, the suspension from wastewater is absorbed on the surface of the biofilm and microalgal cells^24^. Raw sewage contains solid particles such as sand, clay, sludge, mineral crystals, sparingly soluble salts and others, which are retained on the surface of the biofilm due to the EPSs^13,50^. Adsorption of the mineral fraction increases ash content in biomass and decreases the Higher Heating Value of the substrate containing microalgae. Microalgae may contribute more ash in biomass (mean of 30%) compared to terrestrial plants (mean of 7%), which reduces the calorific value^51,52^. The decrease in HHV of the pine bark and the content of Volatile Matter after the experiment (Table 2) may provide indirect evidence for the development of biofilm that removes inorganic solid fraction from wastewater. The bark from reactor B, in which a more abundant growth of algae on the substrate was observed, was characterized by a slightly greater decrease in HHV and VM. This confirms the hypothesis that along with the growth of biomass, the efficiency of removing the suspension from wastewater increases.

**Table 2:** Higher Heating Value and content of Volatile Matter in crude pine bark (control bark) and after the proliferation of microalgal biomass in PBRs A (bark-A) and B (bark-B).

Samples	Parameters			
	Higher Heating Value (MJ kg^−1^)	SD	Volatile Matter (%)	SD
Control bark	22.00	±0.06	98.81	±0.13
Bark-A	21.31	±0.63	95.37	±0.22
Bark-B	20.92	±0.67	93.46	±0.06

Data are shown as the mean (n = 3) with value of standard deviation (SD).

The HHV of pine bark decreased compared to the control sample by more than 3% for PBR A and more than 5% for PBR B. A similar percentage decrease was noted for VM. Therefore, the content of Volatile Matter in microalgal biomass significantly affects its calorific value. The concentration of lipids in the cells of microalgae, which in inversely proportional to the rate of growth and nutrient concentration, may also be responsible for reducing the calorific value of biomass^1,24,51^. The proliferated biomass can be a valuable raw material for energy recovery, for example in the process of composting or fermentation^24^, despite its growth decreasing the energy value of the substrate used. Pine bark with microalgal biomass can be used for bio-oil extraction and production of pyrolysis char with high heating value. Bio-oil obtained from pyrolysis of microalgal biomass has HHV ~ 29 MJ kg^−1^ ^53^, whereas the higher heating value of pyrolysis char from bark is> 30 MJ kg^−1^ ^54^.

The use of sewage in the cultivation of microalgae entails many advantages, such as lower production and operational costs, the possibility of obtaining abundant biomass and sewage treatment. There will also be issues requiring further research, such as e.g. optimal wastewater flow rate, appropriate light intensity and its effective use, as well as CO_2_ supplementation. These factors affect the economic aspects of the construction and operation of technological systems for the cultivation of microalgae^16^. The material of a substrate for the immobilization of biomass has a great importance for the cultivation system and currently no universal substrate for this purpose exists^7^. Pine bark, despite its many advantages, causes an intensification of the color of treated sewage, due to the release of natural pigments and humic acids^29^. In the future, the PBRs can operate in wastewater treatment plant as an additional element of the treatment system. Due to the use of artificial light, the reactors can be independent of the day and night cycles. It is also possible to use additional external light to illuminate the outer layers of the pine bark. A reduction of the costs of microalgae cultivation in a biofilm can also be achieved by replacing pure CO_2_ with a gas mixture containing this compound, for example flue gases or biogas^6^. Pine bark as a substrate for immobilization of algal biomass can also be used in open tanks (artificial or natural) during the algal bloom season, and then the development of biofilm will depend on weather conditions. However, this utilization of the pine bark requires further studies.

## Conclusion

The effectiveness of separation of microalgae depends to a large extent on the density of biomass, thus the use of a solid substrate for the development of biofilm which contains microalgae seems to be an effective solution in order to reduce the costs of cultivation. Moreover, the biofilm with microalgal biomass contributes to a significant improvement in the quality of wastewater in terms of nitrogen, phosphorus and suspended solids concentrations. Pine bark is suitable as a substrate for the harvesting and separation of algal biomass and is also a natural, cheap and easily available material. The HHV and VM content in organic substrate may constitute indirect indicators of biofilm growth during wastewater treatment. Further efforts are required in the search for a substrate for the immobilization of algae, as well as other ways to reduce the cultivation costs in order to increase the utilization of this type of biomass.
